# Formaldehyde scavengers function as novel antigen retrieval agents

**DOI:** 10.1038/srep17322

**Published:** 2015-11-27

**Authors:** Craig T. Vollert, Wilna J. Moree, Steven Gregory, Steven J. Bark, Jason L. Eriksen

**Affiliations:** 1 Department of Pharmacological and Pharmaceutical Sciences, University of Houston, Houston, TX 77204, USA; 2 Biology and Biochemistry, University of Houston, Houston, TX 77204, USA

## Abstract

Antigen retrieval agents improve the detection of formaldehyde-fixed proteins, but how they work is not well understood. We demonstrate that formaldehyde scavenging represents a key characteristic associated with effective antigen retrieval; under controlled temperature and pH conditions, scavenging improves the typical antigen retrieval process through reversal of formaldehyde-protein adduct formation. This approach provides a rational framework for the identification and development of more effective antigen retrieval agents.

In laboratory and clinical settings, aldehyde-based fixation is one of the most widely used approaches to preserve biological tissues for later study. Over the last two decades, a variety of antigen retrieval (AR) techniques have been developed to address the loss of sensitivity associated with aldehyde-based fixatives, ranging from enzymatic digestion^1^ to heat-induced epitope retrieval (HIER)^2^; the most commonly used protocol involves heat and sodium citrate^3^. Although various forms of AR have been developed, the selection of an appropriate protocol has largely remained an empirical process^4^. In this study, we monitored the effect of various AR conditions on formaldehyde treated peptide standards in solution using MALDI mass spectrometry. Based on our findings, we postulate that AR is driven by disassociation of formaldehyde adducts from proteins and the use of formaldehyde scavengers can facilitate this process. We then used mass spectrometry to screen aldehyde scavenger agents unknown in AR for their ability to remove the formaldehyde adducts on model peptides and subsequently tested these compounds in AR of formaldehyde-fixed tissue.

Due to its reported ability to cross-link primary amino groups and other functional groups in proteins, formaldehyde is a common fixative used to preserve tissue for histology. Depending on the amino acid sequence, peptides can undergo a variety of different chemical modifications after formaldehyde fixation which can be divided into three types: (1) methylol groups, (2) Schiff-bases, and (3) methylene bridges^5^. The initial chemical modifications are reversible, and include methylol groups and Schiff-bases, while the formation of methylene bridges are irreversible and develop over longer periods of fixation^6^.

The reversible methylol groups and Schiff-bases are the primary adducts associated with typical formaldehyde fixation^5^ and therefore are likely to represent the principal targets for AR. It is widely thought that the breaking of formaldehyde adducts by heat is the first and most important step in AR^7^,^8^,^9^, while low pH has also been described as an important co-factor for AR techniques^10^,^11^. To evaluate the underlying mechanisms associated with effective AR, and with the goal of uncovering other factors that could improve AR, we analyzed formaldehyde adduct formation on two model peptides induced by formaldehyde treatment, using MALDI-TOF mass spectrometry under different heat and pH conditions, analogous to pre- and post-AR. Angiotensin 1 and adrenocorticotropic hormone (ACTH) (18–39) were chosen as model peptides because of the multiple sites for formaldehyde adduct formation (Angiotensin I has 2 His, 1 Tyr, 1 Arg and N-terminus while ACTH (18–39) contains 1 Lys, 1 Arg, 1 Tyr, 1 Asn, and N-terminus) (Supplementary Fig. S1). MALDI-TOF was only used as a screening tool to determine whether agents should be considered for further screening in the AR experiments, and was not used for quantitation.

Treatment of Angiotensin 1 (*m/z* 1296 Da) (Supplementary Fig. S2) with formaldehyde demonstrated complete conversion to mono- and di-formyl adducts (*m/z* 1308 and 1320 Da respectively). After heating, we observed conversion of the di-adduct group back to the mono-adduct group and lower level conversion to the original Angiotensin 1, suggesting that the formaldehyde adduct formation was reversible. We tested the effect of different pH ranges 3, 7, and 10 on the equilibria of formaldehyde adducts. Significant regeneration of unreacted angiotensin 1 (*m/z* 1296 Da) was observed at pH 3 (Supplementary Fig. S3) but not at pH 7 or pH 10, indicating that the reversal of formalin adducts is pH dependent. These data are consistent with the known mechanism of aldehyde-amine reactions which exist in a state of reversible equilibria and heat and lowering the pH shift this equilibrium towards liberation of formaldehyde into solution.

These observations suggested to us that formaldehyde scavenging agents could act as effective AR agents by binding to and preventing protein-disassociated formaldehyde from reattaching to other proteins after liberation and thereby shifting the equilibrium from the protein adducts towards the original native proteins. To validate these predictions, we identified a set of structurally-unrelated formaldehyde scavenging agents that were able to reverse peptide formaldehyde adducts, as analyzed by MALDI TOF mass spectrometry. 2-Imidazolidinone, ascorbic acid, and tris(hydroxymethyl)aminomethane (tris), all significantly reverse formaldehyde fixation of Angiotensin 1 demonstrated by reduction of the mono-methylene (*m/z* 1308 Da) and di-methylene adduct (*m/z* 1320 Da) and regeneration of the native species (*m/z* 1296 Da) compared to water alone (Fig. 1). MALDI-TOF was only used to monitor ratios between peak intensities of di-methylene, monomethylene and native angiotensin I within each sample and compared to the ratios in formaldehyde treated angiotensin 1. Since retrieval agents can affect crystallization and ionization efficiency of each sample differently, peak intensities of different samples should not be compared.

It is assumed that reversible formaldehyde protein adducts that form during protein fixation with formaldehyde can be transferred from one protein to another. We assessed with MALDI-TOF whether formaldehyde scavengers could prevent this exchange of these adducts between proteins during liberating conditions such as heat. We incubated ACTH (Supplementary Fig. S4) with formaldehyde-treated-myoglobin and heated to 95 °C for 1.5 h. After heating ACTH in the presence of formaldehyde-treated-myoglobin, we observed the addition of a mono-methylene group (*m/z* 2477 Da), indicating formaldehyde adducts transition from formaldehyde-treated-myoglobin to ACTH. In the presence of ascorbic acid, this mono-methylene adduct group was absent, suggesting ascorbic acid prevented the exchange of formaldehyde adducts between proteins^12^. These results also demonstrate that MALDI-TOF mass spectrometry can provide a screening tool for detecting the reversal of formaldehyde labeling. Although the use of MALDI-TOF is effective in determining positive reversal of formaldehyde adduct formation, we did not observe that MALDI-TOF correlated quantitatively with effectiveness of AR. Therefore, final confirmation of reagent effectiveness in AR requires direct analysis in formalin-fixed tissue samples.

We evaluated the effectiveness of the same set of structurally-diverse formaldehyde scavengers as AR reagents. Staining the neurovasculature in formaldehyde-fixed paraffin embedded tissue is a well-known problem that is not amenable to retrieval with traditional AR agents such as sodium citrate^12^,^13^. Traditionally, the infusion of substances such as India ink, lectins, and DiL into an intact vasculature have been used for visualization of the neurovasculature. Collagen IV is a major basement membrane marker and is a highly formaldehyde-sensitive protein that exhibits minimal immunoreactivity in fixed adult brain tissue (Supplementary Fig. S5)^12^. Using a formaldehyde-sensitive collagen IV antibody, we compared the effectiveness of formaldehyde scavengers to traditional AR agents in the fixed adult brain tissue and found that the diverse formaldehyde scavengers all function as effective AR agents, and allow for the detection of collagen IV (Fig. 2; Supplementary Fig. S6) through elimination of formaldehyde adducts; in contrast, sodium citrate was not effective in AR for collagen IV and was ineffective at reversing protein formylation under the conditions examined (Supplementary Fig. S7). Consistent with the reported pH sensitivity of aldehyde-amine reactions, we found that collagen IV immunoreactivity was only observed under acidic (pH 3), but not under neutral or basic (pH 7 or 10), conditions (Supplementary Fig. S8). Although the anti-collagen IV antibody was used in this study because of its sensitivity to formaldehyde-fixation, AR retrieval using these agents allowed for the robust detection of other vascular antigens that can be masked by formaldehyde fixation, such as claudin 5 (Supplementary Fig. S9). Finally, the AR agents we have described are compatible with both immunofluorescence and HRP-based staining techniques with chromogens such as DAB (Supplementary Fig. S10).

In summary, our findings describe a novel mechanism of action for the reversal of formaldehyde fixation by AR agents; this mechanism provides a rational framework for the prediction and screening of novel AR agents that is based upon the use of mass spectrometry as a screening method. Consistent with the literature, we show that most formaldehyde adducts are highly reversible and exist in an equilibrium state (Fig. 3A). Common variables in typical AR techniques, such as heat and low pH, shift this equilibrium towards liberation of the formaldehyde adducts. We identify formaldehyde scavenging as a novel facilitator of AR; scavengers serve to capture liberated formaldehyde and inhibit the released formaldehyde from exchange between proteins, shifting the equilibrium further towards the native proteins and thus providing a basis for the reversal of formaldehyde fixation observed in AR (Fig. 3B). While the approach we have described does not explain other AR mechanisms that have been reported in the literature^14^, our work provides a clear basis for the use of heat and pH in AR, and offers a rational approach to identify new and effective AR reagents based upon their ability to scavenge formaldehyde.

## Methods

Methods and associated references are available in the online version of the paper.

## Methods and Materials

### Animals

Adult (6 mo.) non-transgenic C57BL6/J mice were used in this study. All experimental protocols were approved by the University of Houston Institutional Animal Care and Use Committee. The methods were carried out in accordance with the approved guidelines.

### Tissue Processing

Animals were euthanized with CO_2_ and brains were harvested. Brains were either fixed with formaldehyde or a formaldehyde-free fixative. For formaldehyde fixation, transcardial perfusion with heparinized (2.5 IU/mL) saline followed by 30 ml of 4% (wt/vol) paraformaldehyde in 0.1 M PBS (pH 7.4) was used. Brains were then dissected and post-fixed in 20 ml of 4% paraformaldehyde for 48-72 hr at 4 °C. After fixation brains were processed for paraffin sections (10 μ). For formalin-free fixation, brains were submersed in Accustain (Sigma Aldrich, St. Louis, MO) for three days and then transferred to 70% ethanol solution and stored at 4 °C. Brains were then processed for paraffin sections (10 μ).

### Antigen Retrieval

Paraffin sections were deparaffinized in xylene and rehydrated through graded alcohols. For all antigen retrieval experiments, a Decloaking Chamber (Biocare Medical, Concord, CA) was used for heating. For sodium citrate, slides were heated in 10 mM citrate buffer (pH 6.0) initially at 80 °C and peaked at 125 °C for 45 s and then cooled back down to 80 °C for a total of 45 min. Pepsin AR treatment followed protocol reported by Franciosi *et al.*^15^ For formaldehyde scavengers (2-imidazolidinone, ascorbic acid, and tris) pH was adjusted to 3-4 and slides were initially heated to 80 °C then raised to 95 °C, held for 45 min, then cooled back down to 80 °C for a total of 60 min.

### Immunofluorescence

Tissues were deparaffinized using xylene, graded ethanol baths and rehydration in water. Following rehydration, immunofluorescence was performed on hemibrain sections using the Type IV Collagen antibody (anti-Collagen IV, 1:500, Cosmo Bio USA, Carlsbad, CA) that recognizes the basal lamina of blood vessels and Claudin 5 (1:200, Sigma-Aldrich, St. Louis, MO), a tight-junction protein. Sections were incubated with biotinylated anti-rabbit goat antibody (Vector Laboratories, Burlingame, CA) and visualized using an avidin-linked fluorophore (Vector Laboratories). Slides were imaged using standard epifluorescence conditions using an Olympus IX61 DSU confocal microscope.

### Mass Spectrometry

MALDI-TOF mass spectrometry was performed on an AB Sciex 4800 MALDI-TOF/TOF mass spectrometer using AB Sciex 4000 Series Data Explorer control and processing software (V3.7.1 Build 1, AB Sciex). Samples (0.7 μl) were combined with 0.7 μl of a saturated solution of α-cyano-4-hydroxycinnamic acid (Sigma Aldrich) dissolved in 50% acetonitrile in water with 1% formic acid and spotted onto a 96-well AB Sciex MALDI plate. Each sample was deposited on two different spots. Each spectrum acquired was a composite of 250–500 laser shots. Typically 2–3 spectra for each spot were acquired and only one representative spectrum is displayed in the figures.

The MALDI-TOF mass spectrometer was operated in the delayed extraction/reflector mode with an acceleration voltage of 20 kV, a grid voltage setting of 72%, and a 500 ns delay. Positively charged ions were analyzed in reflection mode. External calibration was performed with a mixture of 5 standard peptides at approximately 100 ng peptides per 10 μl of α-cyano-4-hydroxycinnamic acid solution: Des-Arg(9) Bradykinin (*m/z* = 904.4676 Da), Human Angiotensin 1 (*m/z* = 1296.6848 Da), Glu-1-Fibrinopeptide B (*m/z* = 1570.6768 Da), ACTH 1–17 (*m/z* = 2093.0862 Da), ACTH 18–39 (*m/z* = 2465.1983 Da). All peptides were purchased from Bachem.

### Antigen Retrieval Experiments with Model Peptides

For studies using model peptides, 10 μl of a solution of human angiotensin I (DRVYIHPFHL) purchased from Bachem (5 mg/ml water) or ACTH (18–39 clip) (RPVKVYPNGAEDESAEAFPLEF) (5 mg/ml water) was added to 40 μL formalin (pH 6.8). The mixture was kept at 4 °C for 48 h prior to mass spectrometry.

To the formalin treated peptide (2 μL) was added 98 μl of reagent (5% w/v solution); reagents tested were: ascorbic acid, 2-imidazolidinone, and tris (both buffered to pH 3–4). The mixture was heated at 95–98 °C for 45 min. The reaction was monitored by MALDI-TOF at 3 time points (0 min, 20 min, and 45 min). Experiments were repeated at least 3 times and representative spectra are displayed from the 45 min time point.

### AR agent prevents transfer of formaldehyde label from formaldehyde-treated myoglobin to ACTH (18–39)

Equine Myoglobin (200 μg, Aldrich) (Measured *m/z* = 17150.48 Da, average of 7 measurements) was treated with 200 μL formalin (47%, pH 6.8) for 48 h at 4 ^o^C. (Measured *m/z* = 17776.32 Da, average of 7 measurements).

The formylated myoglobin was concentrated with a Vivaspin 500 3 kDa cut-off filter (GE Healthcare), washed with water (250 μL) for six times, lyophilized, and taken up in 200 μL of water.

Human ACTH (18–39) (1 μL from 0.5 mg/ml stock Bachem) was added to the formylated myoglobin solution (20 μL) and 1 μL of water. Similarly Human ACTH (18–39) (1 μL) was added to formylated myoglobin (20 μL) and 1 μL of ascorbic acid (500 mg/mL water). Mixtures were heated at 95 ^o^C for 1.5 h and monitored by MALDI as described above.

## Additional Information

**How to cite this article**: Vollert, C. T. *et al.* Formaldehyde scavengers function as novel antigen retrieval agents. *Sci. Rep.* **5**, 17322; doi: 10.1038/srep17322 (2015).

## Supplementary Material

Supplementary Information

## Figures and Tables

**Figure 1:**
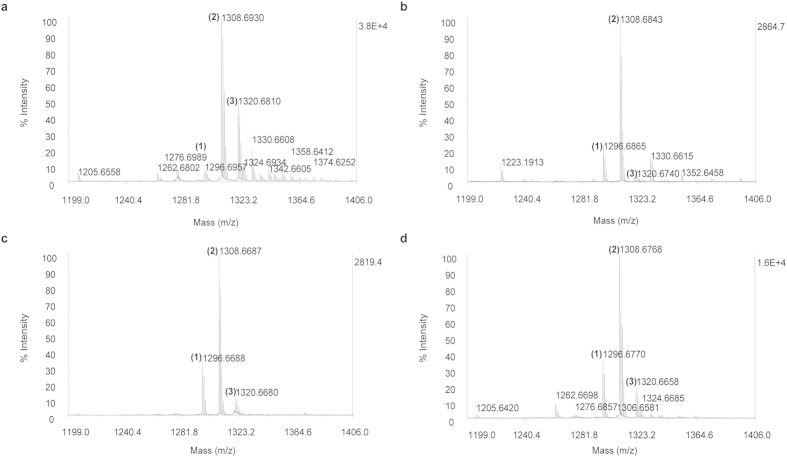
Formaldehyde scavengers reverse formaldehyde modifications of proteins. Retrieval of native angiotensin 1 (*m/z* 1296 Da) with heating was negligible with water alone (pH 5.5) **(a)**, but was clearly evident after heating in the presence of 5% formaldehyde scavenging agents **(b)** 2-imidazolidinone (acidified to pH 3–4), **(c)** ascorbic acid (pH 2–3), and **(d)** tris (pH 3–4). Peaks are labeled as: (1) native angiotensin 1, (2) angiotensin 1 with one methylene unit, and (3) angiotensin 1 with two methylene units.

**Figure 2:**
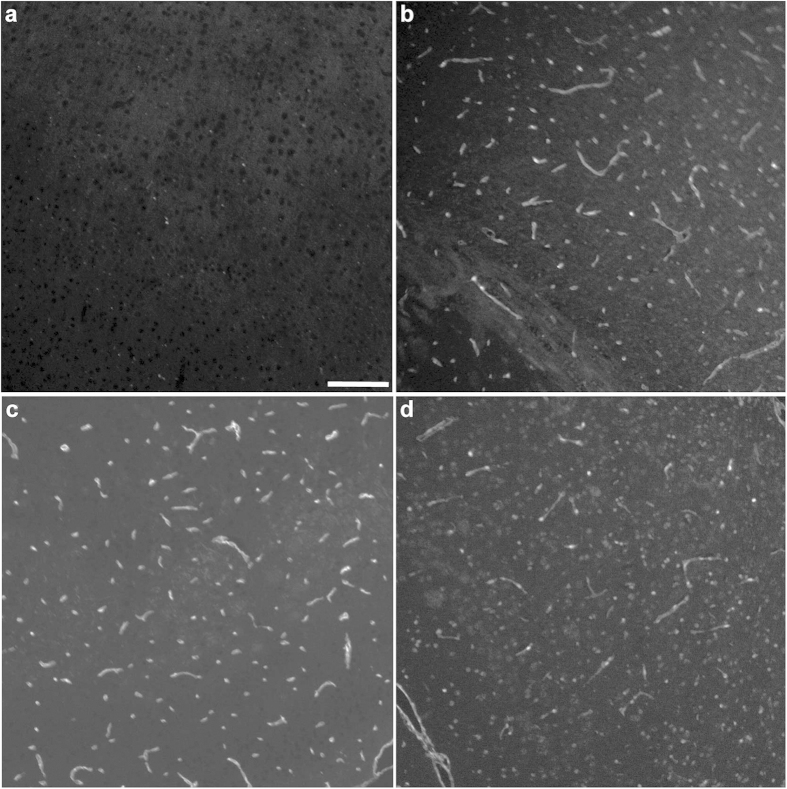
Structurally diverse scavengers function as antigen retrieval agents. Compared with heating in **(a)** water alone, the addition of formaldehyde scavengers, **(b)** 2-imidazolidinone (acidified to pH 3–4), **(c)** ascorbic acid (pH 3–4), and **(d)** tris (pH 3–4), allowed for the robust detection of collagen IV in formalin- fixed adult mouse brain. Scale bar = 50 μm.

**Figure 3:**
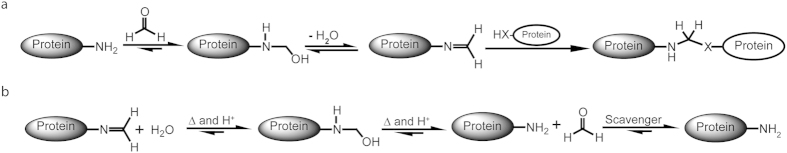
Formaldehyde modifications of proteins during fixation and antigen retrieval. **(a)** During initial fixation, the formation of methylol adducts on amino groups rapidly occurs via methylene glycol. The methylol adducts are then dehydrated yielding Schiff-bases. These processes occur reversibly. Longer-term formaldehyde fixation results in a slow addition to Schiff’s bases leading to formation of irreversible adducts, including methylene bridges and N-terminal formaldehyde cyclization that are unlikely to be affected by antigen retrieval agents. **(b)** Under AR conditions, heat and low pH can lead to rehydration and reversal of the initial reaction. The equilibrium is driven to the right by formaldehyde scavengers; these compounds capture the free formaldehyde that is present in the equilibrium between native protein and methylol/methylene adducts and drive the equilibrium towards native protein.
