# Supplementary material for: Formaldehyde scavengers function as novel antigen retrieval agents
Source: Sci Rep. 2015 Nov 27;5:17322. doi: 10.1038/srep17322 (PMC4661444; doi:10.1038/srep17322)
Supplement: Supplementary Information [file srep17322-s1.pdf]

Supplementary Information

Formaldehyde scavengers function as novel antigen retrieval agents

Craig T Vollert[1], Wilna J Moree[2], Steven Gregory[1], Steven J Bark[2], & Jason L Eriksen*[1]

[1]Department of Pharmacological and Pharmaceutical Sciences, [2]Biology and Biochemistry; University of Houston, Houston, TX 77204, USA.

* To whom correspondence should be addressed. Email: jeriksen@central.uh.edu

Supplementary Figure Legends

Supplementary Figure S1. Structures of model peptides: Angiotensin 1 and ACTH (18-39). Functional groups prone to react with formaldehyde and formaldehyde-protein adducts are color coded; Tyr are in tan and N-containing groups in green. **(a)** Treatment of Angiotensin 1 (DRVYIHPFHL, Monoisotopic *m/z* 1296 Da) with formaldehyde can yield multiple adducts including 1 methylene unit (*m/z* 1308 Da), 2 methylene units (*m/z* 1320 Da), 1 methylene unit and 1 hydroxymethyl group (*m/z* 1238 Da), and 2 methylene units and 1 hydroxymethyl group (*m/z* 1350 Da) (A). **(b)** Addition of formaldehyde to adrenocorticotropic hormone (19-38 clip) (RPVKVYPNGAEDESAEAFPLEF, Monoisotopic m/z 2465 Da) can lead to various adducts including 1 methylene unit (*m/z* 2477 Da), 2 methylene units (*m/z* 2489 Da), 3 methylene units (*m/z* 2501 Da), and 4 methylene units (*m/z* 2513 Da).

Supplementary Figure S2. Heating causes formaldehyde adducts to undergo exchange. (a) Untreated angiotensin 1. **(b)** Treatment of angiotensin 1 with formalin results in a complete conversion to mono- and di-methylene adduct angiotensin 1 (Mono-adduct**:** *m/z* 1308 Da, Di-adduct: *m/z* 1320 Da). **(c)** Heating at 95 °C in water for 45 min resulted in a switch in intensity of the mono- and di-methylene adduct angiotensin 1, but negligible native angiotensin 1 was observed. All reactions were carried out at pH 6.8. Peaks are labeled as: (1) native angiotensin 1, (2) angiotensin 1 with one methylene unit, and (3) angiotensin 1 with two methylene units.

Supplementary Figure S3. Reversal of formaldehyde adduct is pH dependent. Formalin treated angiotensin 1 under different aqueous pH conditions. Retrieval of unreacted angiotensin 1 (*m/z* 1296 Da) was observed at pH 3 **(a)**, but not at pH 7 or 10 **(b and c)**. Peaks are labeled as: (1) native angiotensin 1, (2) angiotensin 1 with one methylene unit, and (3) angiotensin 1 with two methylene units.

Supplementary Figure S4. Formaldehyde scavengers prevent formaldehyde-labeling of ACTH (18-39) by formaldehyde-treated myoglobin. (a) Untreated ACTH (*m/z* 2465 Da) heated in water. **(b)** A small amount of ACTH was converted to mono-methylene adduct ACTH (*m/z* 2477 Da) when heated in the presence of formaldehyde-treated myoglobin (pH 6.8). **(c)** Conversion of ACTH was not observed when heated in the presence of formaldehyde-treated myoglobin and 5% ascorbic acid (pH 2-3). Peaks are labeled as: (1) native ACTH and (2) ACTH with one methylene unit.

Supplementary Figure S5. Collagen IV antibody is sensitive to formaldehyde-fixation in tissue. (a) Collagen IV immunoreactivity is completely eliminated after formaldehyde fixation. **(b)** However, under identical staining conditions and without any antigen retrieval, Collagen IV immunoreactivity is preserved in tissue fixed in a formalin-free fixative.

Supplementary Figure S6. Formaldehyde scavengers, but not traditional antigen retrieval agents, allow the detection of formaldehyde-sensitive epitopes. Collagen IV antibody was used as a formalin-sensitive epitope on coronal sections of formaldehyde fixed mouse brain tissue. Heating in water did not retrieve the **(a)** collagen IV antigen; similar, treatment with **(b)** sodium citrate and **(c)** and pepsin were unable to retrieve the collagen IV epitope. Use of formaldehyde scavenging agents, such as **(d)** ascorbic acid (other agents not shown), successfully retrieved the collagen IV epitope.

Supplementary Figure S7. Sodium citrate does not sufficiently reverse formylation of angiotensin 1. (a) Treatment of angiotensin 1 with formaldehyde at pH 6.8 results in a complete conversion to mono- and di-methylene adduct angiotensin 1 (Mono-adduct**:** *m/z* 1308 Da, Di-adduct: *m/z* 1320 Da**). (b)** Native angiotensin 1 (*m/z* 1296 Da) was weakly retrieved after heating with 5% sodium citrate (pH 8). Peaks are labeled as: (1) native angiotensin 1, (2) angiotensin 1 with one methylene unit, and (3) angiotensin 1 with two methylene units.

Supplementary Figure S8. Detection of collagen IV epitope is pH dependent. Formaldehyde-sensitive Collagen IV antibody was used on coronal sections of formaldehyde fixed mouse brain tissue under different pH conditions. The collagen IV epitope was unmasked when heated at pH 3 in ascorbic acid **(a)**, but not at pH 7 or 10 **(b and c)**. Scale bar = 50 μm.

Supplementary Figure S9. Formaldehyde scavengers allows visualization of Claudin 5 antigens. A formaldehyde-sensitive Claudin 5 antibody was tested on coronal sections of formaldehyde-fixed mouse brain tissue under different AR conditions. Claudin 5 was not detectable using sodium citrate antigen retrieval **(a)** but unmasked when heated in ascorbic acid **(b)**. Scale bar = 50 μm.

Supplementary Figure S10. Formaldehyde scavengers allows visualization of blood vessels with immunoperoxidase staining. Compared with heating in **(a)** water alone, the addition of a formaldehyde scavenger **(b)** ascorbic acid allowed for robust detection of collagen IV using immunoperoxidase staining and visualization with DAB in formalin-fixed adult mouse brain. Scale bar = 50 μm.

a

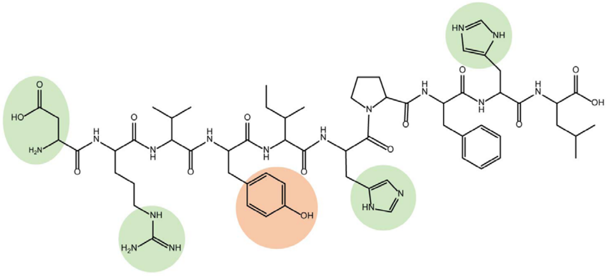

b

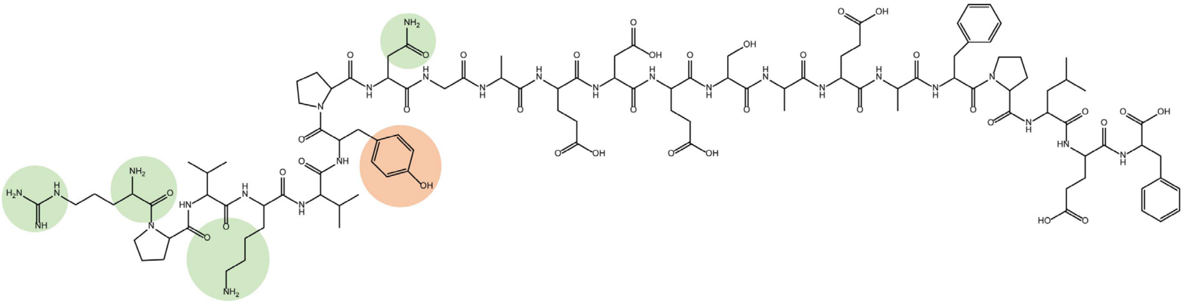

Supplementary Fig. S1

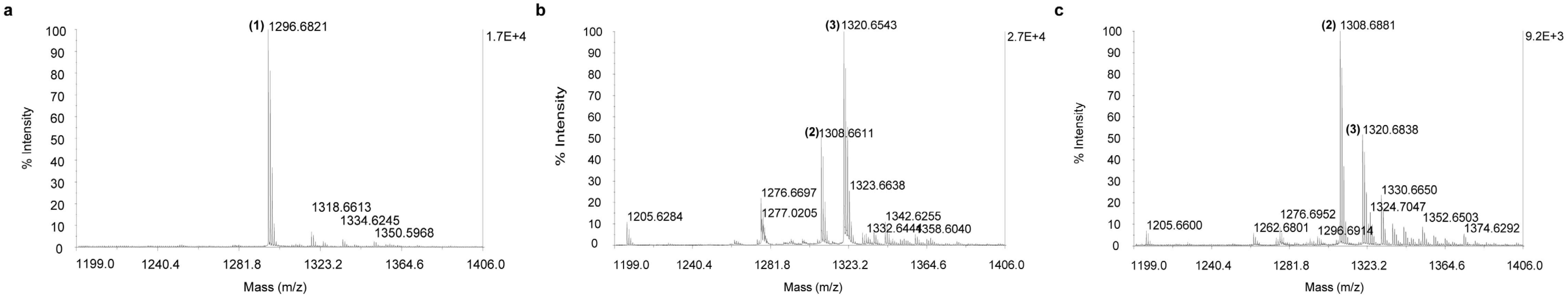

Supplementary Fig. S2

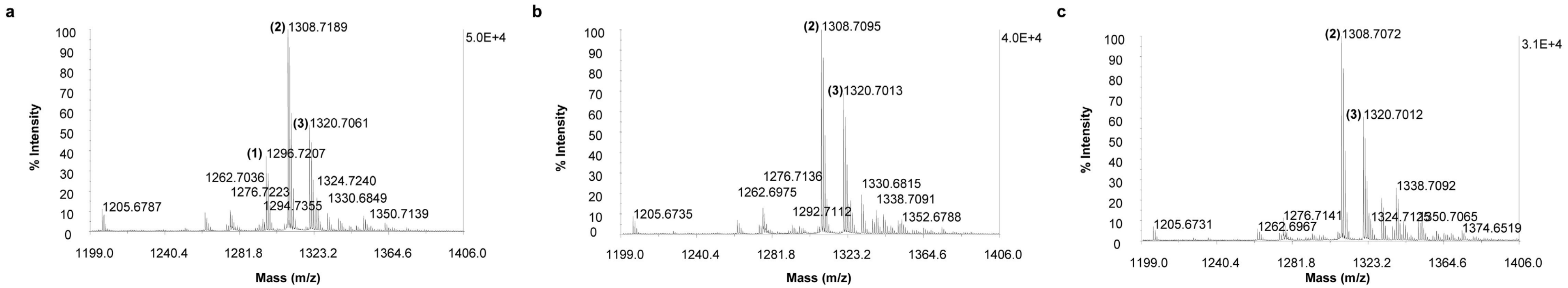

Supplementary Fig. S3

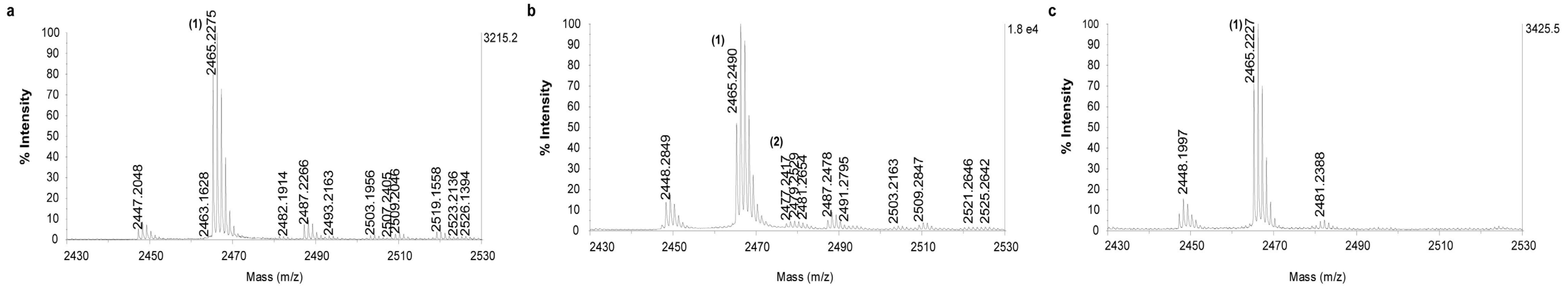

Supplementary Fig. S4

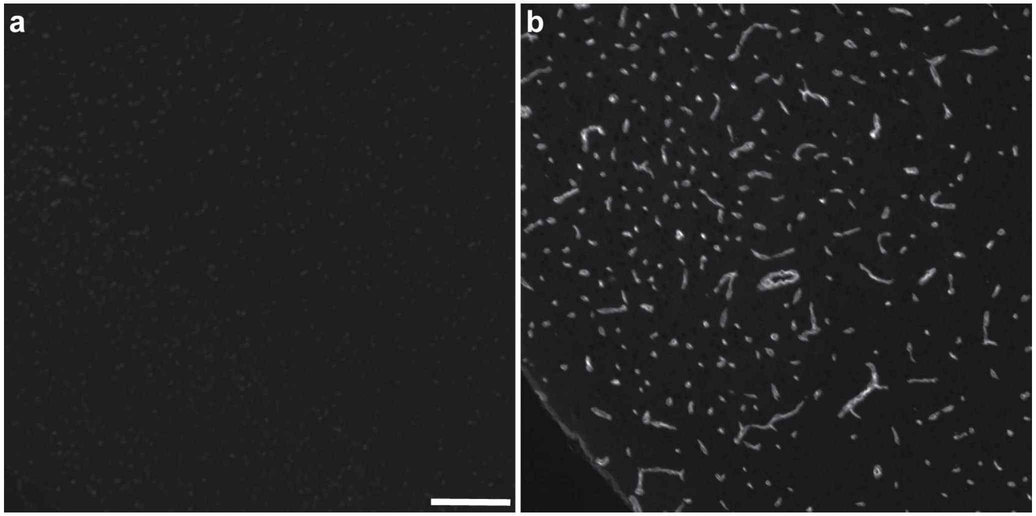

Supplementary Fig. S5

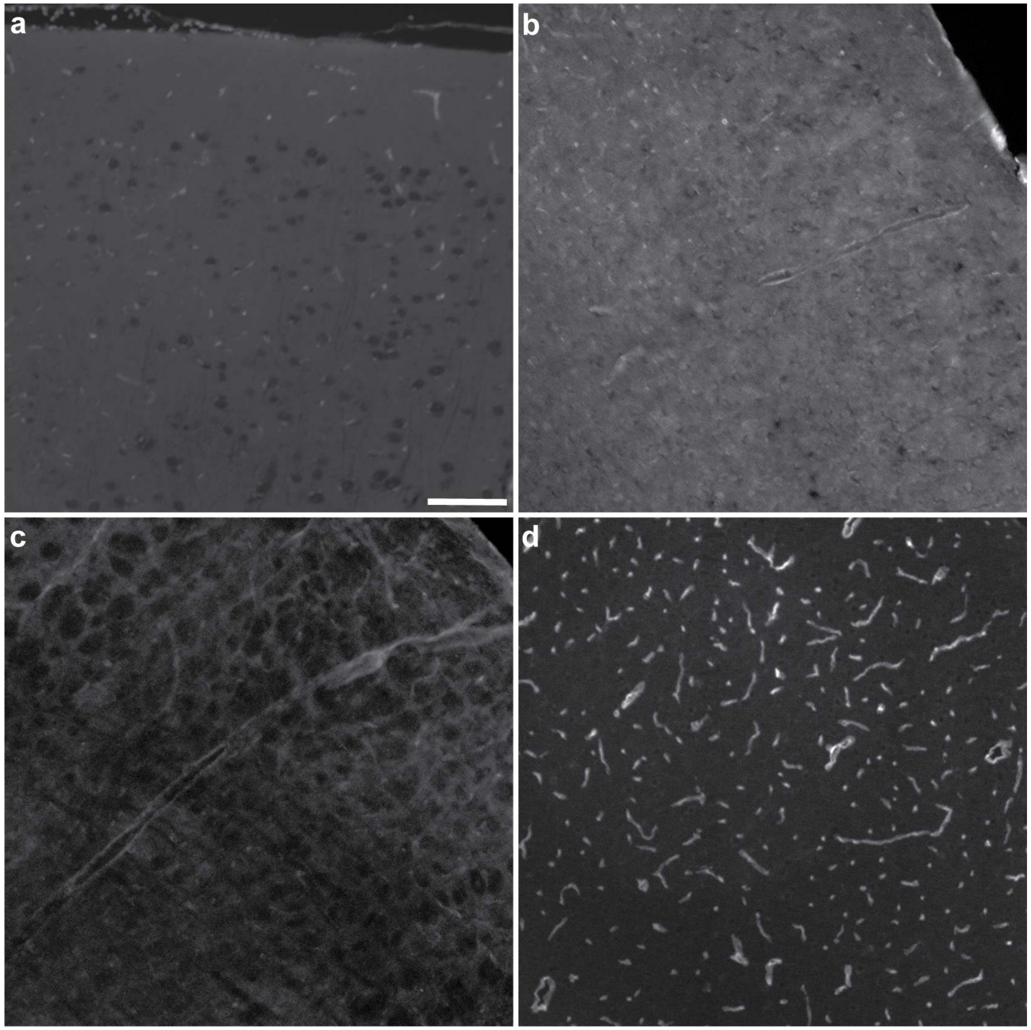

Supplementary Fig. S6

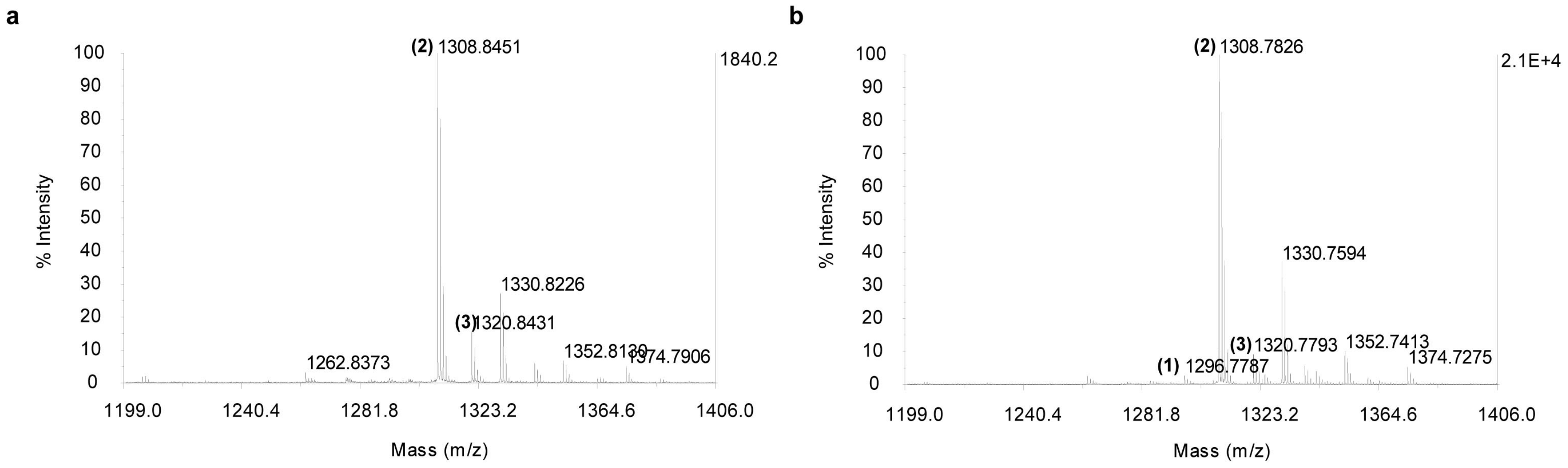

Supplementary Fig. S7

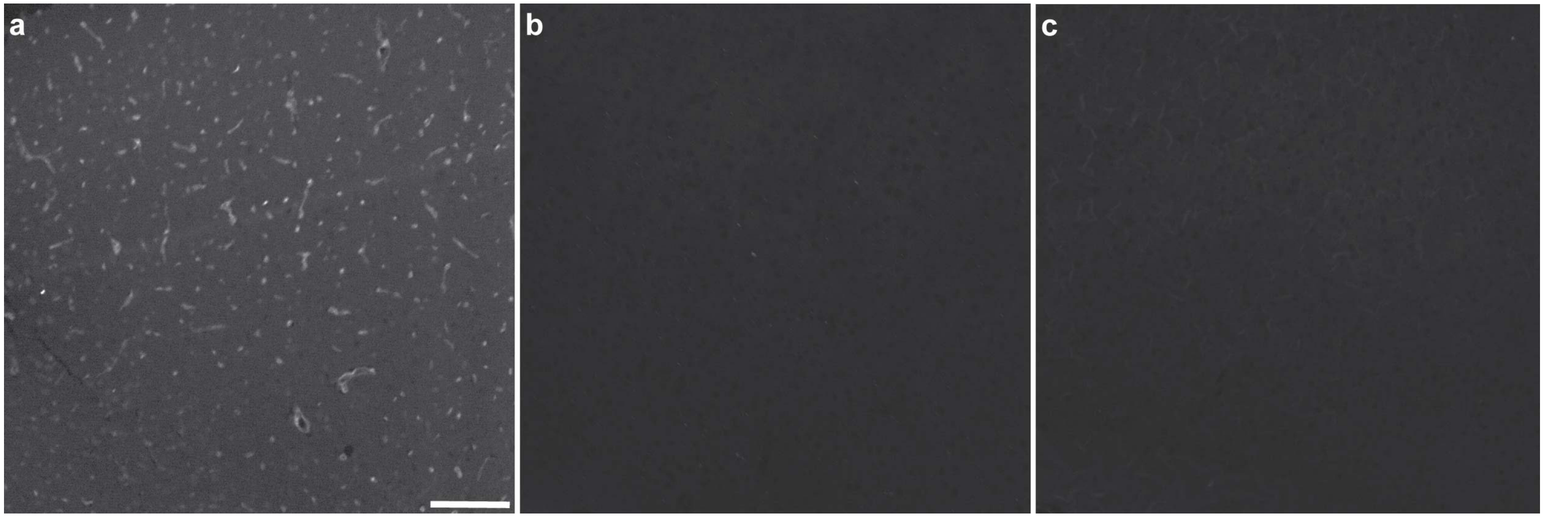

Supplementary Fig. S8

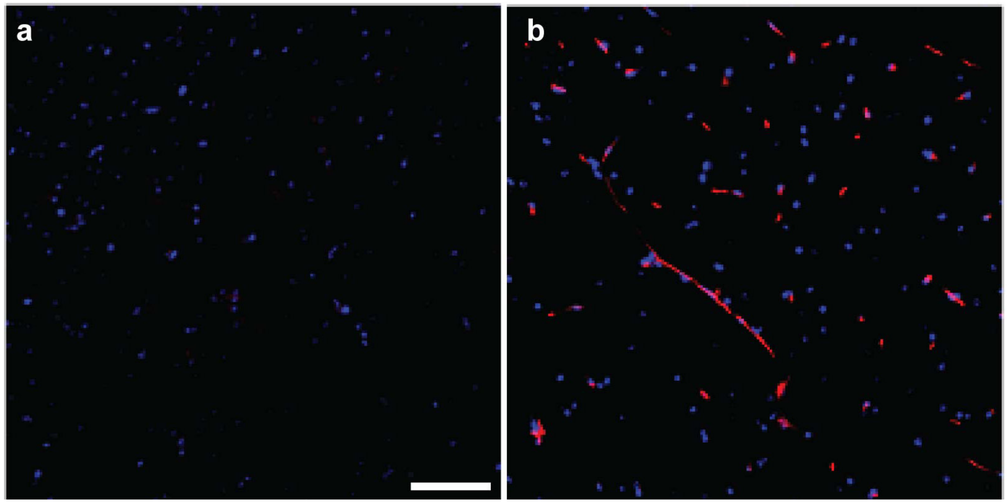

Supplementary Fig. S9

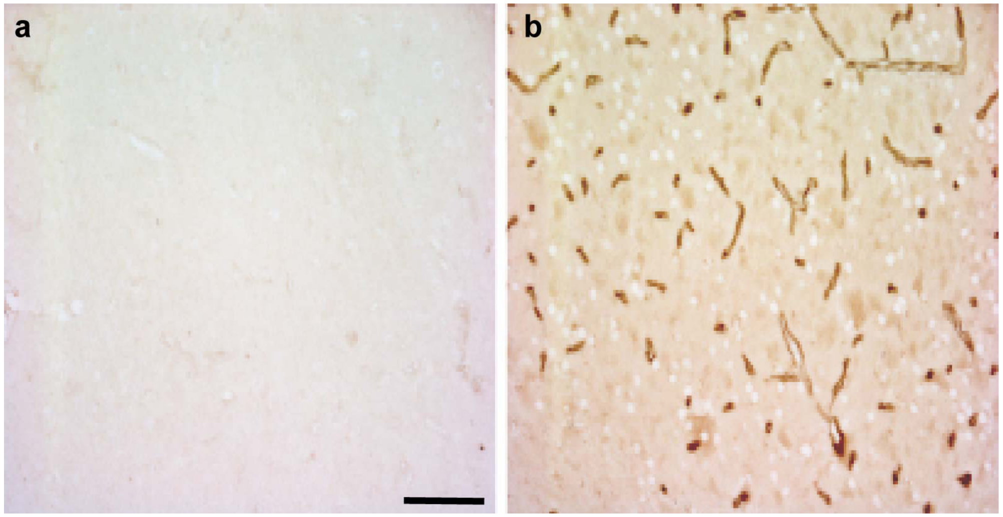

Supplementary Fig. S10
